# Predicting boiling heat flux, heat transfer coefficient, and regimes Non-intrusively using external acoustics and deep learning

**DOI:** 10.1038/s41598-025-08183-z

**Published:** 2025-07-02

**Authors:** Doyeong Lim, Yang Liu, In Cheol Bang

**Affiliations:** 1 Department of Nuclear Engineering, Ulsan National Institute of Science and Technology, Ulsan, Republic of Korea; 2 Department of Nuclear Engineering, Texas A&M University, College Station, TX USA

**Keywords:** Boiling, Acoustics, Deep learning, Heat flux, Heat transfer coefficient, Boiling regime, Engineering, Energy science and technology

## Abstract

Accurate monitoring of boiling heat transfer is critical for the safety and efficiency of high energy-density systems, including data center cooling, nuclear reactors, and industrial boilers. Traditional diagnostic methods relying on intrusive sensors or visual inspection become impractical in harsh industrial environments characterized by high pressures, temperatures, and radiation exposure. In this paper, we propose a non-intrusive diagnostic framework combining externally measured acoustic emission (AE) signals with advanced deep learning techniques. Pool boiling experiments were conducted from natural convection to critical heat flux (CHF), and AE signals were externally collected under various boiling conditions. Through a comprehensive evaluation of hundreds of models, a transformer-based model demonstrated optimal performance, simultaneously predicting key boiling parameters—heat flux, heat transfer coefficient (HTC), and boiling regime—with prediction errors of less than 20% for heat flux and HTC, and over 98% accuracy in boiling regime classification. Further validation on subcooled flow boiling confirmed robust generalizability. Our results reveal that frequency-domain characteristics of AE signals strongly correlate with boiling phenomena, enabling interpretable and reliable diagnostics. This method provides simultaneous prediction of critical boiling parameters without invasive instrumentation, significantly enhancing operational safety and improving reliability in thermal management systems.

## Introduction

Boiling is a highly efficient thermal management mechanism due to its rapid phase transition and substantial latent heat, which enables heat removal at one-order higher heat fluxes than convection^1^. Consequently, boiling heat transfer is integral to various thermal energy systems, ranging from data center cooling and industrial boilers to power plants and nuclear reactors. However, these systems typically operate under high heat flux conditions, requiring accurate real-time monitoring of boiling parameters—such as heat flux, heat transfer coefficient, and boiling regime—to prevent system failures caused by temperature excursions when boiling conditions degrade. Traditional monitoring methods rely predominantly on visual inspection techniques using high-speed^2^ or infrared cameras^3–5^ and internal temperature and void fraction sensors^6,7^. These approaches, while valuable in controlled laboratory environments, become impractical in harsh industrial conditions characterized by high pressure, elevated temperatures, radiation exposure, and limited accessibility^8,9^. Additionally, reliance on empirical correlations derived from local sensor data necessitates conservative safety margins due to inherent uncertainties. Therefore, there exists a critical need for robust, non-intrusive boiling diagnostic methods capable of reliably assessing boiling phenomena and reducing operational uncertainties, thereby optimizing performance, improving safety, and extending the lifetime of critical thermal system components.

To address this need, this study presents a novel acoustic emission-based, deep learning-driven framework for non-intrusive boiling monitoring. Boiling acoustic emission (AE) signals are elastic waves generated by bubble nucleation, growth, and collapse at the external walls of a boiling system^10^. AE sensors can be mounted outside the pressure boundary, avoiding issues related to harsh conditions, radiation, or mechanical constraints. Although previous studies have demonstrated that AE signals can provide valuable insights into two-phase dynamics^11–13^including boiling regimes identification^14–16^heat flux quantification^17^and critical heat flux conditions^18–21^most of these studies have relied on intrusive methods involving hydrophones submerged in fluid or have encountered significant challenges in reliably correlating externally measured AE signals with boiling phenomena. The primary difficulty arises from the high noise levels present in externally measured AE data, which include background disturbances and complex signal interactions induced by solid–fluid^10^. These inherent limitations necessitate advanced techniques capable of effectively extracting boiling performance parameters from AE signals measured externally.

Recent advances in machine learning, and in particular deep learning, offer promising solutions for analyzing complex, noisy data in diverse engineering challenges^22,23^. Deep learning models provide good mapping capability at automatically extracting patterns from high-dimensional, noisy data—precisely the conditions. By training on sufficiently diverse datasets collected under various operating conditions, deep neural architectures can identify meaningful correlations between features and underlying phenomena^24–27^. Moreover, deep learning frameworks can continuously learn and improve as more data become available, potentially adapting to a broad array of industrial scenarios^28–31^.

Despite recent progress in applying artificial intelligence to acoustic emission (AE) signals for boiling diagnostics, previous studies predominantly utilized acoustic sensors submerged within the fluid, such as hydrophones, to predict either boiling regimes^19,32^or heat flux individually^33,34^. For instance, Dunlap et al.^17^ predicted heat flux through acoustic signals from immersed sensors employing Gaussian process regression. However, employing submerged sensors introduces significant practical limitations, particularly in harsh industrial environments characterized by high pressure, elevated temperature, or radiation hazards. successfully predicted heat flux from submerged acoustic sensor data using Gaussian process regression. However, the use of submerged sensors poses significant practical limitations in industrially relevant environments characterized by high pressure, elevated temperature, or radiation hazards, making internal sensor placement impractical or impossible. Alternatively, acoustic signals can also be measured in air using microphones, as demonstrated by Zhang et al.^35^ who utilized microphone-measured acoustic signals combined with deep learning techniques to classify boiling regimes. However, AE signals captured through air suffer substantial signal attenuation, increased background noise interference, and limited sensitivity, restricting their capability to accurately predict quantitative boiling parameters such as heat flux and heat transfer coefficient (HTC). Thus, a key research gap remains in developing non-intrusive, externally measurable methods that can simultaneously predict multiple boiling parameters.

In this study, we introduce a nonintrusive, deep learning-based framework for decoding boiling acoustic signals. We combine state-of-the-art deep learning models—including deep neural networks^22^convolutional neural networks^22,36^transformers^37^and Fourier neural operators^38^—with AE data collected from pool boiling experiments^21^. After identifying an optimal architecture that reliably predicts heat flux, heat transfer coefficients, and boiling regimes, we further assess its robustness by applying it to flow boiling data^39^. This approach represents the first demonstration of externally mounted contact AE sensors integrated with deep learning to simultaneously predict internal heat flux, HTC, and boiling regime, offering a practical and scalable solution for industrial boiling diagnostics.

## Results and discussion

### Acoustics of boiling

**Fig. 1:**
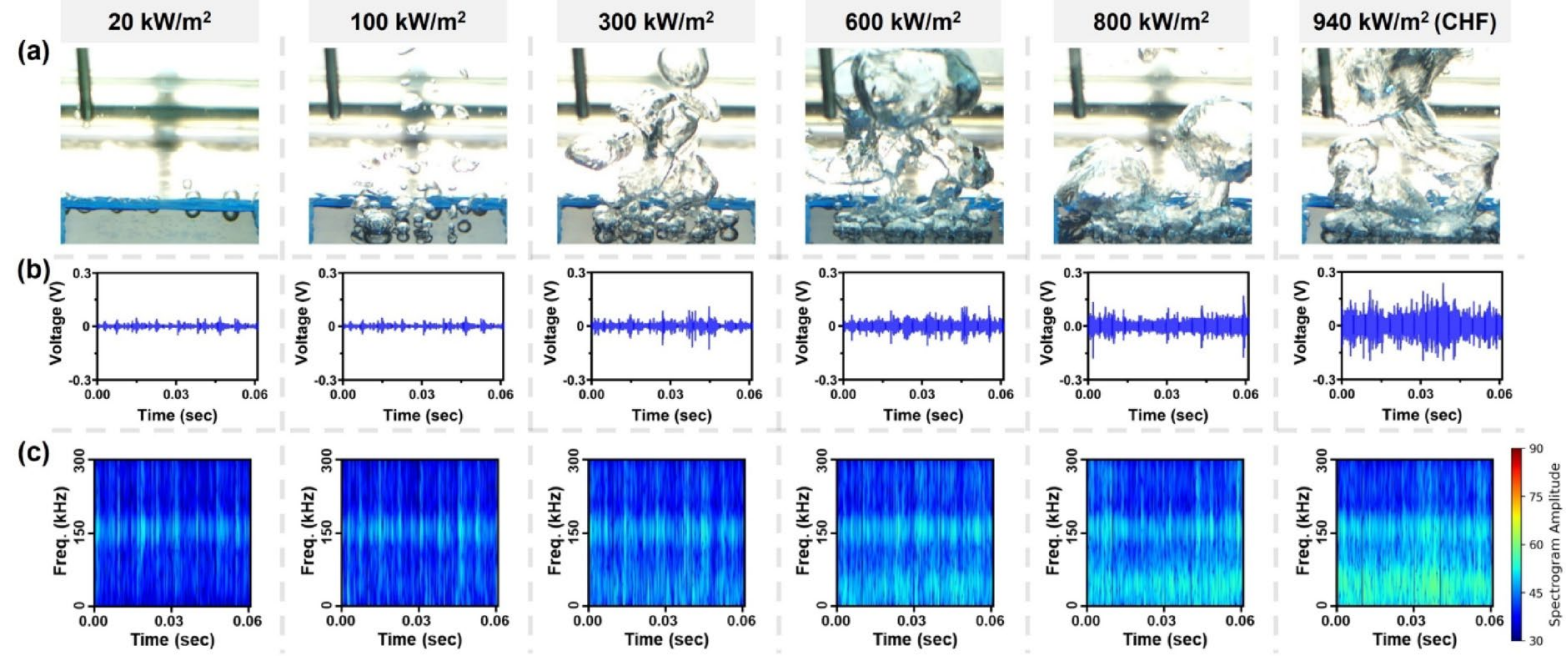
Experimental result: (**a**) high-speed visualization images, (**b**) acoustic emission signals voltage with time, and (**c**) spectrogram converted by STFT with time-frequency domain at each heat flux steps.

AE signals were obtained from pool boiling experiments, with the heat flux increased from natural convection regime, through nucleate boiling, and up to the critical heat flux (CHF). Figure 1(a) presents high-speed visualizations of boiling phenomena, while Fig. 1(b) shows the corresponding AE signals at each step. Figure 1(c) presents spectrograms transformed from the AE signals using the short-time Fourier transform (STFT), providing time-frequency domain information and their intensities from time-intensity domain. As discussed from previous study^21^boiling-induced AE signals arise primarily from the rapid pressure fluctuations that occur during bubble nucleation.

Under natural convection regime (20 kW/m²), distinct AE signals did not appear, because AE generation generally requires a physical event such as bubble nucleation. In contrast, Fig. 1(a) illustrates that at relatively low heat fluxes in the nucleate boiling regime (100–300 kW/m²), isolated bubbles form on the heater. Under these conditions, individual nucleation remains well-separated, allowing the AE signal generation. The most intense AE pulses occur immediately after nucleation, during the bubble’s rapid “inertial” growth phase lasting less than 1 ms^21^. This short, explosive expansion produces an impulse-type AE waveform, with dominant frequencies centered around 10–80 kHz.

As the heat flux increased, isolated bubble nucleation evolved into more intense, collapsed boiling behavior, notably between about 300 kW/m² and 800 kW/m². Boiling-related AE signals commonly appeared below 50 kHz, and their intensity and frequency grew with increasing boiling phenomena. CHF occurred around an average of 912.3 kW/m² (± 27.7 kW/m²), peaking near 940 kW/m², at which point high-frequency signals above 150 kHz were detected. Despite spectral signatures, the intricate, multi-scale relationships between AE signals and boiling parameters are not straightforward to characterize, thereby necessitating a more advanced modeling approach such as deep learning.

### Performance evaluation of deep learning models

**Fig. 2:**
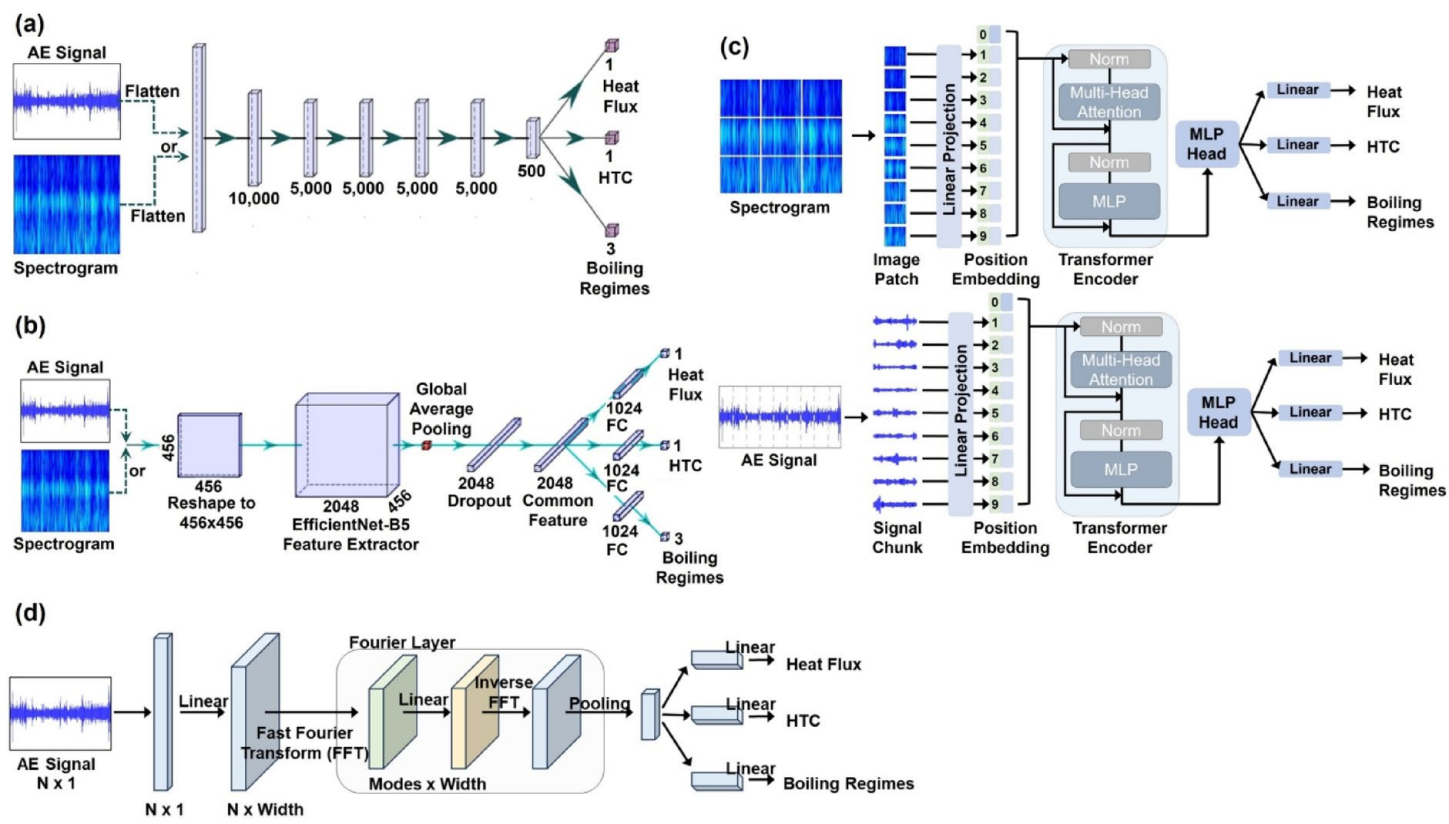
Deep learning models for AE signal-based boiling performance monitoring: (**a**) Deep neural network, (**b**) Convolutional neural network, (**c**) Transformer, (**d**) Fourier neural operator.

This study focuses on the development of deep learning models to predict heat flux, HTC, and boiling regime simultaneously using AE signal data measured externally from the boiling system. Building upon the experimental data acquisition and deep learning methodologies described in the *Methods* section, 245 different models were trained using only 10% of the dataset (randomly selected 1,810 data points out of 18,106). This initial step enabled efficient exploration of a broad range of hyperparameter combinations and model architectures without excessive computational time, thereby allowing us to identify the most promising model candidates. After evaluating models with 10% of the dataset, we then retrained selected models using the complete dataset. As shown in Fig. 2, the models evaluated include four primary architectures: Deep Neural Networks (DNN), Convolutional Neural Networks (CNN), Transformers (TF), and Fourier Neural Operators (FNO), with input features used as either raw AE signals (Signal) or their spectrogram (Spec) for sensitivity analysis (for detailed description, please refer to *Methods* section).

**Fig. 3:**
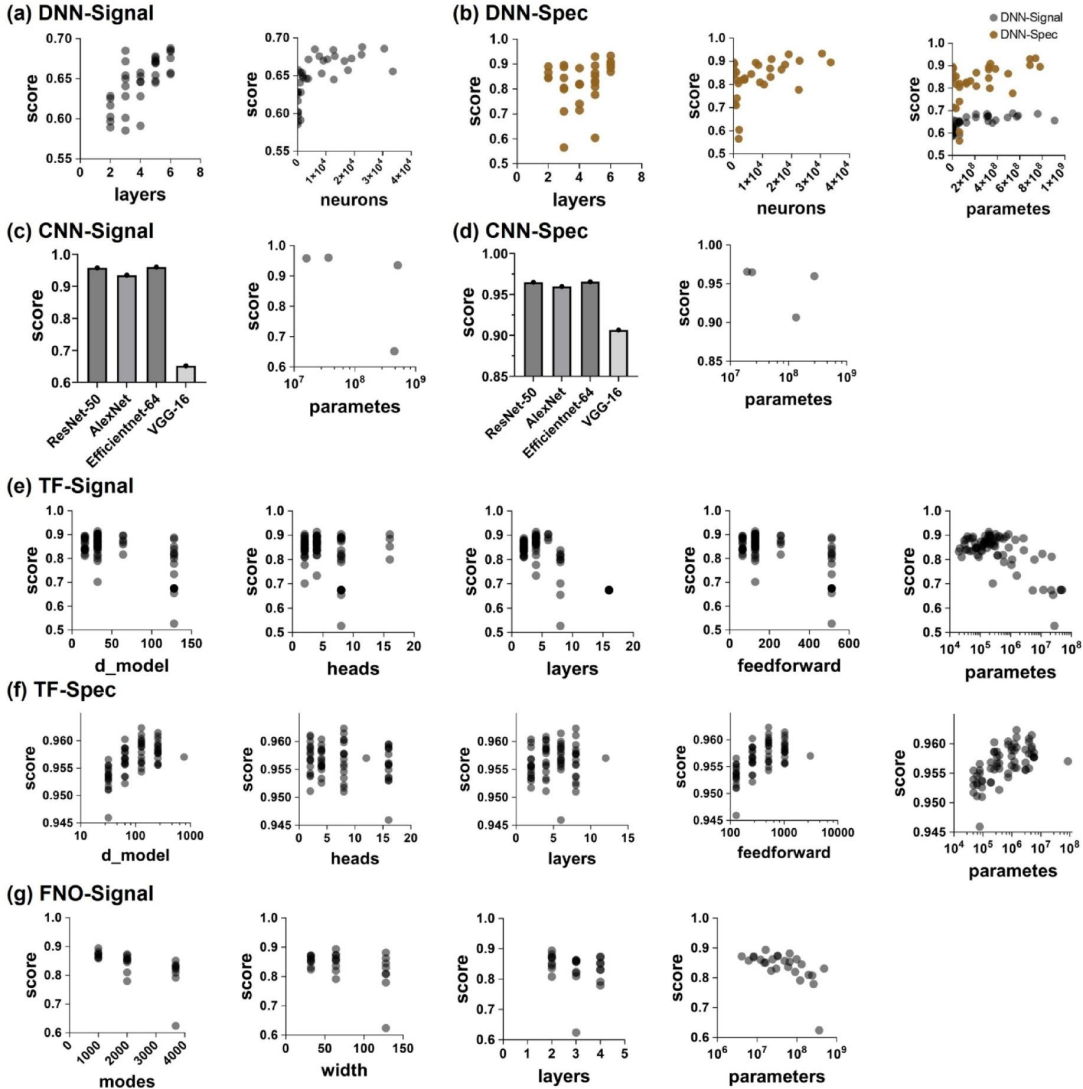
Sensitivity analysis of trained deep learning models with hyperparameter for (**a**) DNN with AE signal input, (**b**) DNN with spectrogram input, (**c**) CNN with AE signal input, (**d**) CNN with spectrogram input, (**e**) Transformer with AE signal input, (**f**) Transformer with spectrogram input, (**g**) FNO with AE signal input.

Figure 3; Table 1 present the results of sensitivity analysis results using a randomly selected 10% subset of the dataset, thereby enabling a systematic comparison of model architectures and hyperparameters for AE-based boiling prediction. Seven configurations were evaluated by varying key hyperparameters (e.g., hidden-layer width, number of convolutional filters, attention heads, and Fourier modes). Each model was assessed using (i) regression metrics such as the normalized root mean square error (NRMSE) for heat flux and HTC, (ii) classification accuracy for the boiling regime, and (iii) an overall ‘score’ computed as the average of (1 − NRMSE for heat flux), (1 − NRMSE for HTC), and classification accuracy. Models using raw AE signals (e.g., DNN-Signal, CNN- Signal, TF- Signal, FNO- Signal) generally required far more parameters to approach similar performance, whereas those using spectrograms (DNN-Spec, CNN-Spec, TF-Spec) consistently achieved superior regression precision and classification accuracy with fewer trainable weights. Notably, CNN-Spec demonstrated strong aptitude for extracting spatial features from the spectrogram, while TF-Spec effectively modeled both temporal and frequency-domain dependencies; both surpassed the FNO-AE approach, which relies exclusively on frequency-domain transformations. These findings underscore the advantages of spectrogram-based representations for robust and efficient prediction of boiling parameters.

In Table 1, optimal deep learning models were derived by inputs (AE signal or Spectrogram) and architectures. Among spectrogram-based models, CNN-Spec achieves an RMSE of 51.0 for heat flux, 2.0 for HTC, and an accuracy of 0.994. TF-Spec shows similarly strong results, with an RMSE of 42.6 for heat flux and an accuracy of 0.963. These findings indicate that convolutional models excel at identifying spatial features in spectrograms, while Transformers effectively model the temporal dependencies, making them highly suitable for acoustic time-frequency data. Although FNO-Signal outperforms DNN-Signal, its RMSE of 218.1 and classification accuracy of 0.919 fall short of CNN-Signal and TF-Signal, implying that integrating Fourier transforms does not surpass the representational power of convolutional or Transformer-based architectures in this application.

**Table 1:** Comparative performance metrics of optimal deep learning models using AE signals and spectrograms on randomly selected 10% of the dataset.

Predictions			Heat flux		HTC		BR
**Model**		Parameters	RMSE	NRMSE	RMSE	NRMSE	Acc.
**DNN-Signal**	[10000; 5000; 5000; 2000; 500; 250]	536.8 M	353.4	0.375	10.0	0.329	0.711
**CNN-Signal**	EfficientNet-b4	36.7 M	52.5	0.056	1.7	0.056	0.992
**TF-Signal**	[32, 4, 4, 32]	0.067 M	75.6	0.080	2.8	0.092	0.989
**DNN-Spec**	[10000; 5000; 5000; 5000; 5000; 500]	739.9 M	**40.9**	**0.043**	**1.2**	**0.036**	0.966
**CNN-Spec**	EfficientNet-b4	19.4 M	51.0	0.054	2.0	0.066	**0.994**
**TF-Spec**	[128; 8; 8; 512]	1.4 M	42.6	0.045	1.5	0.051	0.963
**FNO-Signal**	[1000; 64; 2]	32.8 M	218.1	0.232	6.5	0.214	0.919

### Comprehensive evaluation of optimal models

Following the initially derived models, each optimal model was retrained using the entire dataset comprising 18,106 data points. This comprehensive analysis allowed for a more robust assessment of model performance on the prediction tasks of heat flux, HTC, and boiling regime classification. The retrained models’ results are summarized in Table 2 with comparative performance metrics.

The results clearly indicate that spectrogram-based models, including DNN-Spec, CNN-Spec, and TF-Spec, outperformed their signal-based counterparts across all tasks. Among the spectrogram-based models, TF-Spec demonstrated the best overall performance. Specifically, it achieved the lowest RMSE for heat flux (22.6) and HTC (0.77), along with the lowest NRMSE values of 0.024 for heat flux and 0.026 for HTC. Furthermore, TF-Spec achieved a boiling regime classification accuracy of 0.976, confirming its superior generalization capability. CNN-Spec followed closely, achieving slightly lower RMSE and NRMSE values in heat flux and HTC prediction but the highest boiling regime classification accuracy of 0.983. These results present the effectiveness of convolutional architectures in capturing spatial patterns in spectrogram data, while the Transformer-based approach excels at modeling temporal dependencies.

**Table 2:** Full dataset training results for optimal models.

Predictions	Heat flux		HTC		BR	Score
Model	RMSE	NRMSE	RMSE	NRMSE	Acc.	
**DNN-Signal**	365.3	0.390	12.09	0.411	0.364	0.521
**CNN-Signal**	2730.7	2.908	115.33	3.921	0.608	−1.407
**TF-Signal**	443.1	0.472	9.96	0.339	0.709	0.633
**DNN-Spec**	38.9	0.041	1.20	0.041	0.958	0.958
**CNN-Spec**	36.2	0.039	1.14	0.039	**0.983**	0.969
**TF-Spec**	**22.6**	**0.024**	**0.77**	**0.026**	**0.976**	**0.975**
**FNO-Signal**	204.1	0.217	7.12	0.242	0.824	0.788

In contrast, signal-based models, including DNN-Signal, CNN-Signal, TF-Signal, and FNO-Signal, struggled to achieve comparable performance. The DNN-Signal model, with its large parameter count, demonstrated the weakest performance, with a heat flux RMSE of 365.3 and HTC RMSE of 12.09. Its boiling regime classification accuracy of 0.364 was also the lowest among all models, indicating significant limitations in extracting meaningful features directly from raw AE signals. Similarly, CNN-Signal, despite leveraging the powerful EfficientNet-b4 architecture, showed a surprising degradation in performance when applied to signal data, with a heat flux RMSE of 2730.7 and an HTC RMSE of 115.33. This suggests that convolutional architectures are less effective at capturing raw signal characteristics compared to frequency-domain features provided by spectrograms.

Among the signal-based models, the Fourier Neural Operator (FNO-Signal) demonstrated some performance improvements over traditional DNN-Signal models, achieving a heat flux RMSE of 204.1 and a boiling regime classification accuracy of 0.824. However, its performance remained substantially below that of the spectrogram-based models. These findings reinforce the importance of effective input representations, as raw AE signals lack the structured features necessary for accurate and consistent predictions.

The results from the full dataset training provided the clear superiority of spectrogram-based models with the TF-Spec model, emerging as the optimal choice for predicting heat flux, HTC, and boiling regimes. In addition, with only 1.44 million parameters, the TF-Spec model supports fast inference—approximately 20–30 ms per 224 × 224 spectrogram on an RTX 3090 GPU—enabling real-time throughput of 30–50 FPS, making it suitable for timely and continuous monitoring.

**Fig. 4:**
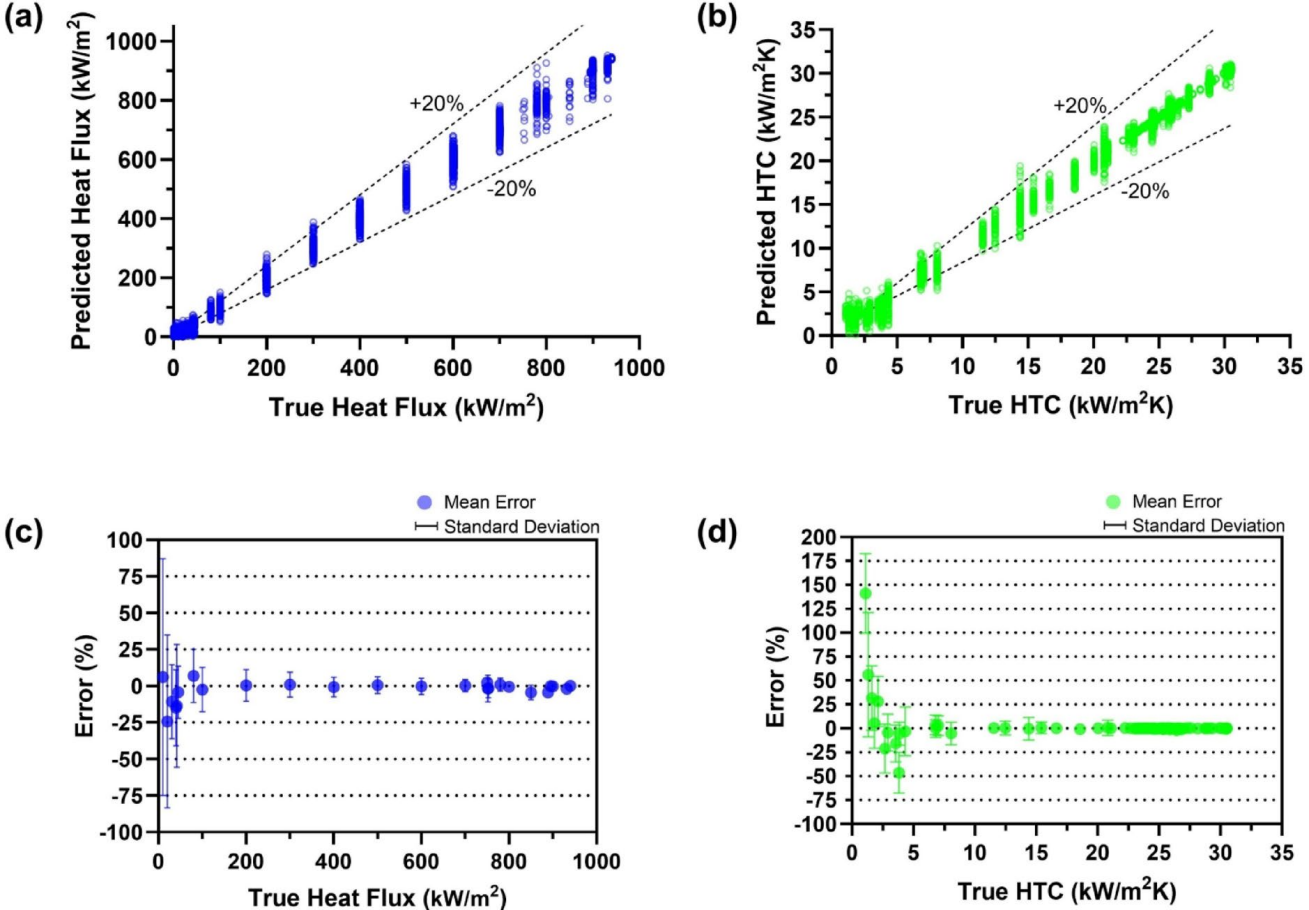
TF-Spec model prediction results for (**a**) heat flux, (**b**) heat transfer coefficient with 20% error lines, (**c**) mean prediction error of heat flux, and (**d**) mean prediction error of heat transfer coefficient. Error bars represent standard deviation.

Figure 4 presents predicted heat flux and HTC inferred by TF-Spec model. Figure 4(a) and (b) compare these predictions with true values, while Fig. 4(c) and (d) illustrate the percentage error distributions for these predictions. The results demonstrate that the TF-Spec model achieves a high level of accuracy, with predictions falling within a ± 20% error margin in nucleate boiling and CHF regime. This level of accuracy is comparable to the inherent uncertainties stemming from the AE sensor and the instrumentation used in measuring boiling parameters. In particular, the R15a-type contact AE sensor (MISTRAS Group) employed in this study exhibits directionality and frequency-response variability of up to ± 1.5 dB, translating to about ± 17.4% amplitude (voltage) uncertainty. When combined with the ± 1.4% heat flux uncertainty and ± 2.2% HTC uncertainty (refer to Table 3 in *Methods* section), achieving ± 20% accuracy in overall predictions is arguably within the expected range of measurement fidelity. A key driver of the relatively high uncertainty in AE sensing is the complex, multi-path nature of acoustic wave propagation through solid materials and boundary interfaces—along with variability in sensor orientation and resonance. If the sensor uncertainty can be further reduced, the accuracy of heat flux and HTC predictions is likely to be proportionally improved.

Despite this performance, notable deviations occur in the low heat flux (< 40 kW/m²) and HTC (< 10 kW/m²K), particularly within the natural convection regime, which exhibits significant errors as illustrated in Fig. 4(c) and (d). The errors are attributed to the absence of boiling on the heater, which results in distinct AE signals beyond the background noise. Consequently, the lack of distinguishable features in the AE signals makes accurate predictions challenging in these conditions. Practically, this result suggests that the proposed AE-based approach is most reliable in the nucleate boiling regime, where substantial boiling produces distinct AE signatures. For applications in which the heat flux is below the onset of nucleate boiling, the sensor signals may not offer sufficient information to infer heat transfer parameters accurately.

**Fig. 5:**
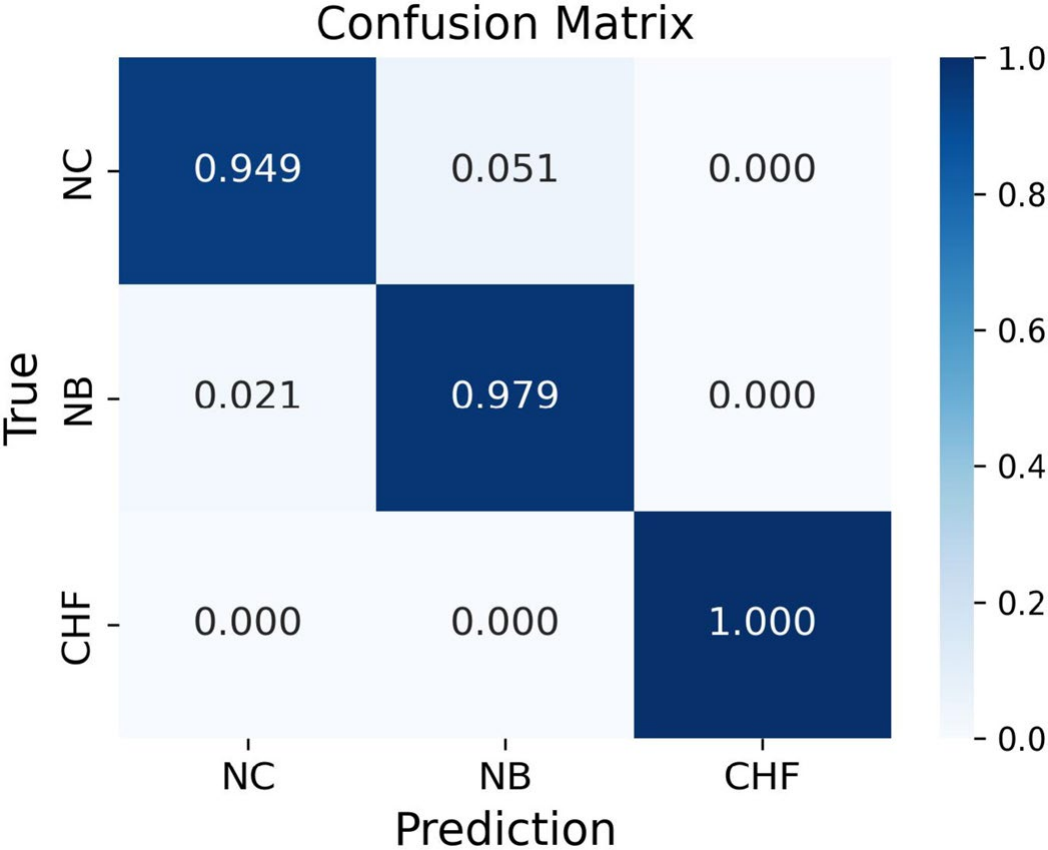
Confusion matrix for boiling regimes classification (TF-Spec model).

For boiling regime classification, Fig. 5 presents the confusion matrix for the TF-Spec model’s classification performance, encompassing three regimes: natural convection (NC), nucleate boiling (NB), and CHF. The matrix reveals the high predictive accuracy of the model with overall classification. Unlike the regression of heat flux and HTC, the absence of a characteristic AE signal in NC makes it easy to distinguish regimes. The TF-Spec model achieved an accuracy of 94.9% for the NC regime, 97.9% for the NB regime, and 100% accuracy for the CHF regime. These results demonstrate the model’s exceptional ability to identify the boiling regime. The NC regime exhibited a 5.1% misclassification rate, where a small fraction of instances was incorrectly classified as NB. This can be attributed to the transitional nature of AE signals in the lower boundary of the NB regime, which may overlap with background noise in the NC regime. Similarly, the NB regime showed a minor 2.1% misclassification rate into NC. Notably, no misclassifications occurred for the CHF regime, reflecting the model’s ability to effectively capture the highly distinctive AE signatures associated with CHF conditions.

**Fig. 6:**
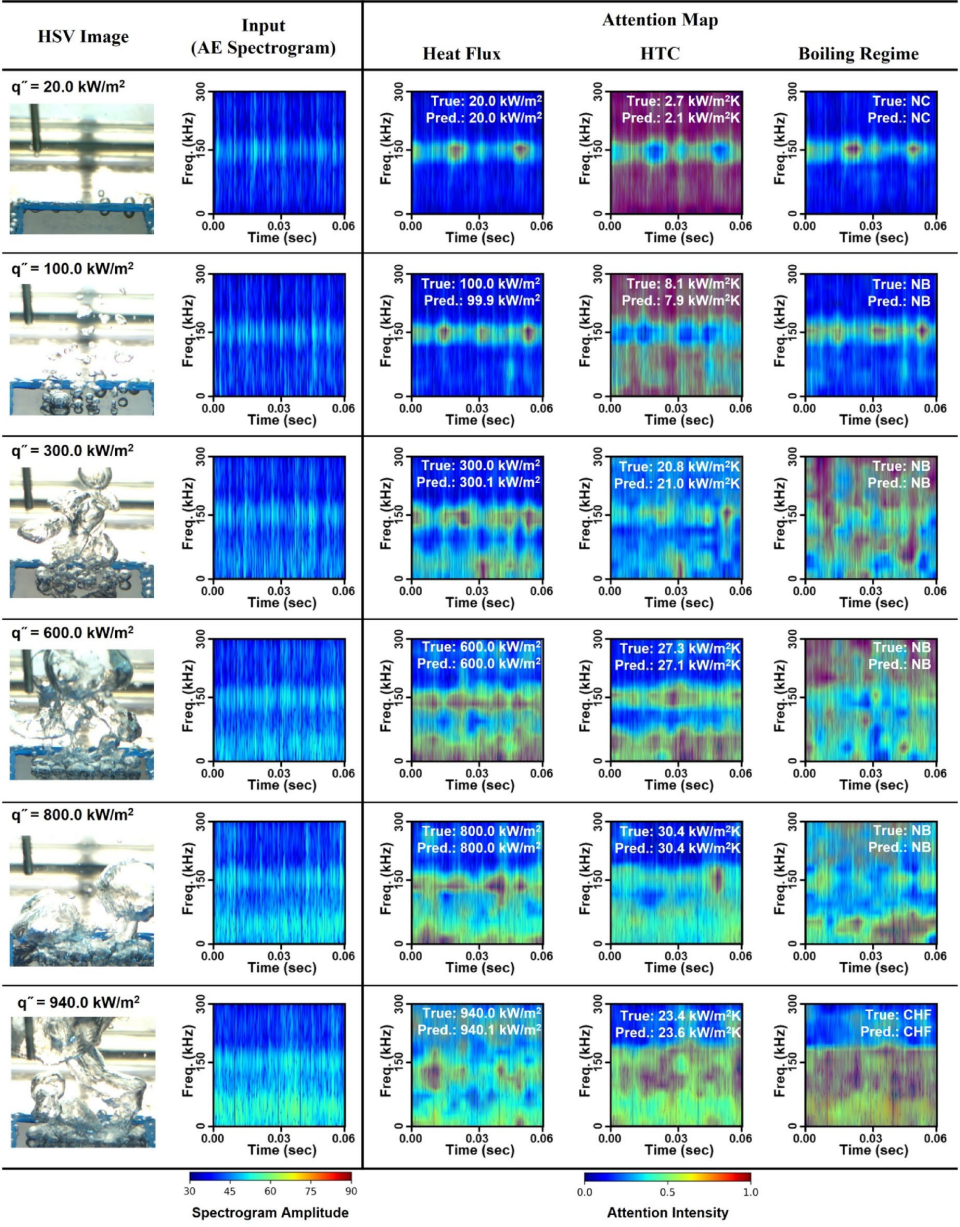
The observed boiling state, the spectrogram used as model input, and the model’s attention distributions for predicting heat flux, HTC, and boiling regime—along with true versus predicted values. Color scales indicate either spectrogram amplitude or attention intensity, highlighting the frequency–time regions most influential for each prediction.

For investigating the inference attributes of TF-Spec model, Fig. 6 provides a comprehensive visualization of the TF-Spec model’s attention map to predict heat flux, HTC, and boiling regimes. Figure 6 is organized into six rows representing distinct heat flux conditions, consisting of five elements: high-speed visualizations, spectrograms, and three attention maps. The second column presents the spectrograms used as input to the TF-Spec model. In the NC regime (20 kW/m²), the spectrogram shows low-intensity signals with minimal variation. As the heat flux increases into the NB regime (100–800 kW/m²), the spectrograms display progressively higher signal intensities with distinct patterns at various frequencies, reflecting the increased acoustic activity from bubble nucleation and collapse. At 940 kW/m² (CHF), the spectrogram exhibits broad, high-intensity signals spanning the entire frequency range, indicative of chaotic boiling and significant AE signal generation.

Figure 6 illustrates how the TF-Spec model allocates its attention based on boiling conditions. In the nucleate boiling regime (100–800 kW/m²), the model predominantly focuses on AE signals below 80 kHz, which are strongly associated with bubble nucleation. Conversely, under natural convection conditions at 20 kW/m², where boiling does not occur, the model tends to rely on signals around 150 kHz—this frequency corresponds to the sensor’s resonance and captures subtle vibrations that are insufficient for accurate heat flux estimation, as reflected by the larger prediction errors observed in Fig. 4. At the critical heat flux (CHF) condition (940 kW/m²), the model integrates information across the entire frequency range (0–300 kHz). This broad focus is likely due to the rapid temperature rise and the resultant micro-cracking in the heater, which generate additional high-frequency components that are crucial for precisely predicting heat flux in CHF conditions.

For HTC prediction, the attention patterns differ notably from those observed for heat flux. Under natural convection (20 kW/m²), instead of focusing on the 150 kHz band, the model utilizes all frequency ranges except the 150 kHz region, suggesting that the aggregated information from other frequencies better correlates with HTC under these conditions. As the boiling process enters the nucleate regime, where AE signals primarily appear below 80 kHz, the model reinforces its focus on these low-frequency bands to infer HTC. Under CHF conditions, the model expands its attention over the entire frequency range up to approximately 170 kHz, capturing not only the acoustic signals from boiling activity and bubble collapse but also the effects of heater degradation.

For boiling regime classification, the attention maps in natural convection show that the model primarily focuses on the 150 kHz region—consistent with the absence of lower-frequency bubble activity. This high-frequency focus confirms that the lack of characteristic boiling signals is a key indicator of the natural convection regime. During nucleate boiling, while it is anticipated that the model would predominantly concentrate on low-frequency signals (below 80 kHz), the observed attention spans a broader frequency range. This expanded focus not only reflects the model’s effort to capture subtle transitions and variations within nucleate boiling but also cautiously suggests that there may be additional, previously unreported acoustic features contributing to the boiling process. In the CHF regime, the attention is distributed across almost the entire spectrogram. Here, the model captures widespread, high-intensity AE signatures resulting from both boiling activity and heater-related disturbances. This comprehensive attention across the full frequency spectrum enables the model to accurately distinguish the CHF regime from other boiling conditions.

### Cross-System validation

The adaptability and robustness of deep learning models are essential for their application to various thermal systems. While the TF-Spec model has shown exceptional accuracy in predicting heat flux, HTC, and boiling regimes under pool boiling conditions, its applicability to systems with different operational characteristics has not yet been evaluated. Flow boiling, characterized by boiling with fluid flow by the pump, introduces additional complexities such as flow-induced bubble dynamics, convective heat transfer, and variations in AE signal patterns. Evaluating the TF-Spec model using flow boiling data provides an opportunity to test its ability to generalize to thermal systems with distinct physical and dynamic behaviors (detailed explanation of flow boiling experiment, please refer to the *Methods* section).

**Fig. 7:**
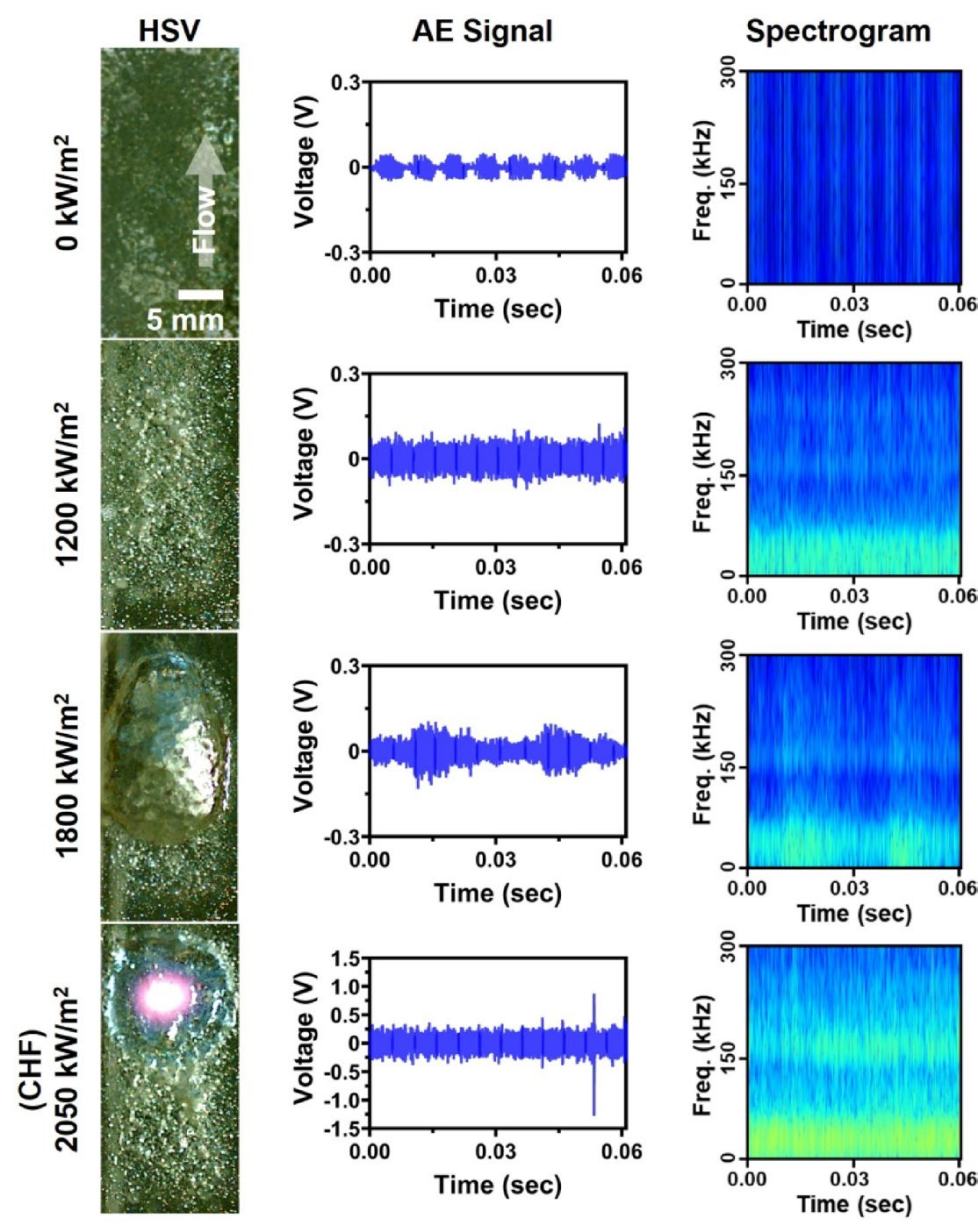
Subcooled flow boiling experimental results with visualization images, AE signals, and spectrograms at heat flux of 0 kW/m^2^, 1200 kW/m^2^, 1800 kW/m^2^, and 2050 kW/m^2^.

**Fig. 8:**
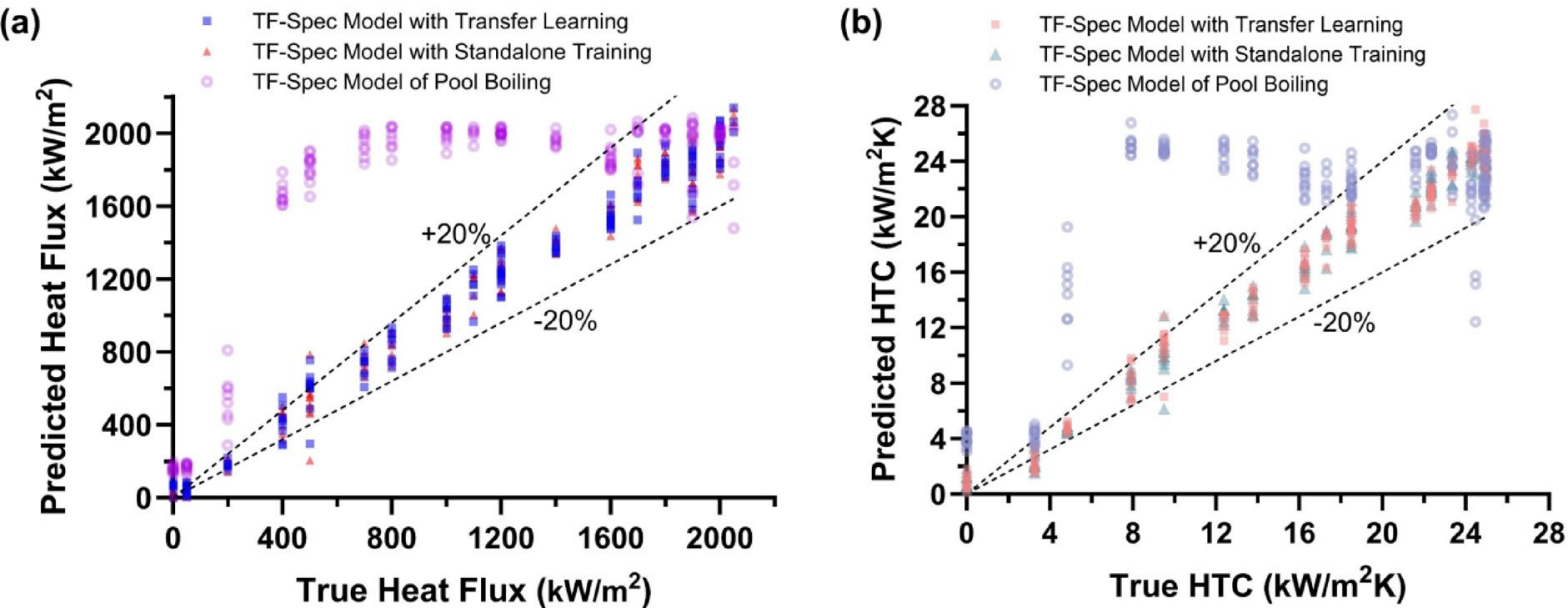
Subcooled flow boiling experimental loop for AE signal acquisition.

To investigate this case study, AE signal data were collected from a subcooled flow boiling experiment, as illustrated in Figs. 7 and 8. The experiment conducted vertical upward flow boiling within a closed-loop system utilizing deionized water under atmospheric pressure. AE signals were recorded as the heat flux increased, capturing transitions from the convection regime to nucleate boiling and to CHF. The boiling behaviors observed under flow boiling conditions were notably different from those of pool boiling, particularly in terms of AE signal characteristics and bubble dynamics, due to the influence of fluid flow. Specifically, the forced flow causes more frequent and smaller bubble detachments, often resulting in overlapping acoustic signals that differ from the distinct, impulse-type waveform observed in pool boiling, for example, pump-induced turbulence is described at spectrogram vertical line pattern in 0 kW/m^2^. Consequently, these factors create different acoustic environments than pool boiling, highlighting the importance of specialized model adaptation (e.g., transfer learning). From the experiment, A total of 854 data points was collected, comprising 680 for training and 174 for testing, allowing for a robust analysis of the model’s generalization and predictive capabilities in this complex environment.

**Fig. 9:**
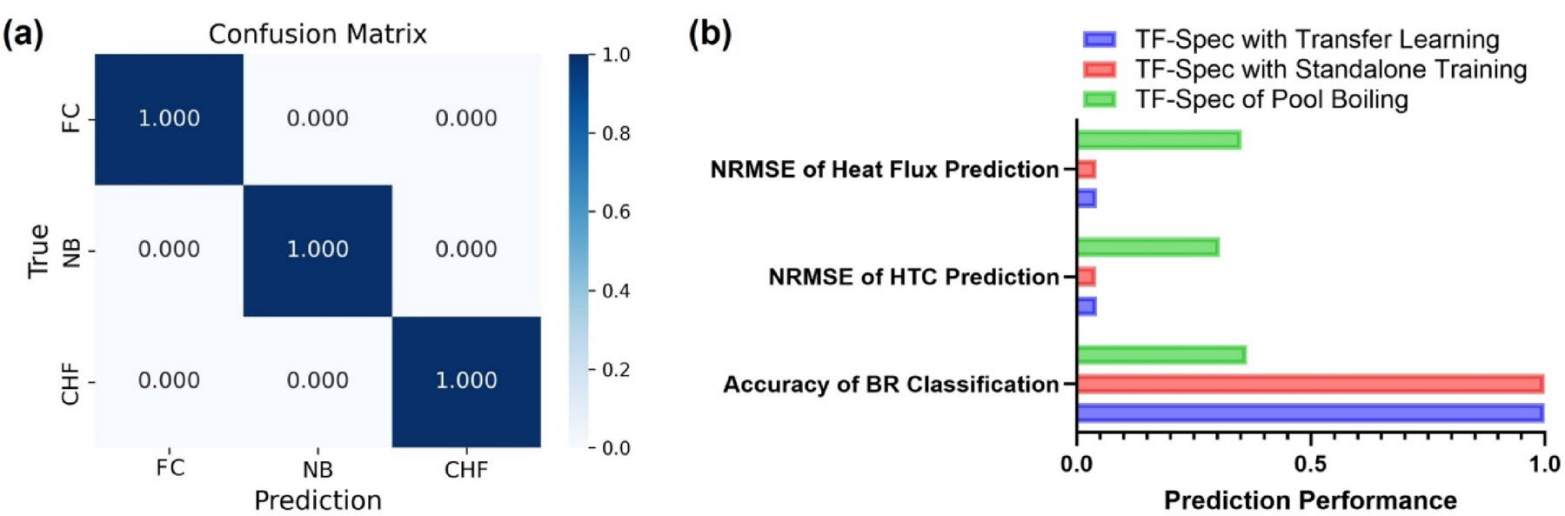
TF-Spec Model prediction results for flow boiling; (**a**) heat flux prediction, and (**b**) heat transfer coefficient with 20% error lines.

By training the models, Fig. 9 illustrates the performance of three TF-Spec model configurations in predicting heat flux and HTC. The models tested include: (1) TF-Spec with Transfer Learning, where the model pretrained on pool boiling data was further fine-tuned using flow boiling data, (2) TF-Spec Model with Standalone Training, which was trained solely on flow boiling data from scratch, and (3) TF-Spec Model of Pool Boiling, which was trained exclusively on pool boiling data and directly applied to flow boiling predictions without any additional training.

In Fig. 9(a), the predicted heat flux is plotted against the true heat flux values, and in Fig. 9(b), the predicted HTC is compared with the true HTC. Both panels include a ± 20% error margin to evaluate the accuracy of the models across the entire operational range. The TF-Spec Model of Pool Boiling exhibited significant prediction errors across much of the heat flux range, particularly in the convection and nucleate boiling regimes (below 1,600 kW/m²). This highlights the limitation of directly applying a model trained on pool boiling data to flow boiling systems, where distinct physical mechanisms, such as subcooling of working fluid that leads to smaller bubble size and rapid contraction, and flow-induced bubble dynamics. However, at heat flux values above 1,600 kW/m², corresponding to the merged bubble and CHF regimes, the model’s predictions improved, reflecting some overlap in AE signal patterns between high-heat-flux conditions in pool and flow boiling. In contrast, both the Transfer Learning and Standalone Training models demonstrated exceptional predictive accuracy, with nearly all predictions falling within the ± 20% error margin. These models successfully captured the distinct AE signal patterns associated with flow boiling, providing their ability to adapt to the unique characteristics of flow boiling systems. The Transfer Learning approach achieved slightly better alignment with true heat flux values, indicating that pretraining on pool boiling data provided a slight advantage in model performance.

For HTC predictions, a similar trend is observed. The TF-Spec Model of Pool Boiling showed large prediction errors across most of the HTC range, failing to capture the influence of flow-induced convective heat transfer on AE signal characteristics. On the other hand, the Transfer Learning and Standalone Training models exhibited highly accurate predictions, with almost all points within the ± 20% error margin. The results from these models closely resemble the performance observed in the original pool boiling experiments, suggesting that both training approaches are effective in learning the AE signal-to-HTC relationship in flow boiling. As with heat flux prediction, the Transfer Learning model displayed slightly superior performance, likely due to the enriched feature representations derived from its pretraining phase.

**Fig. 10:**
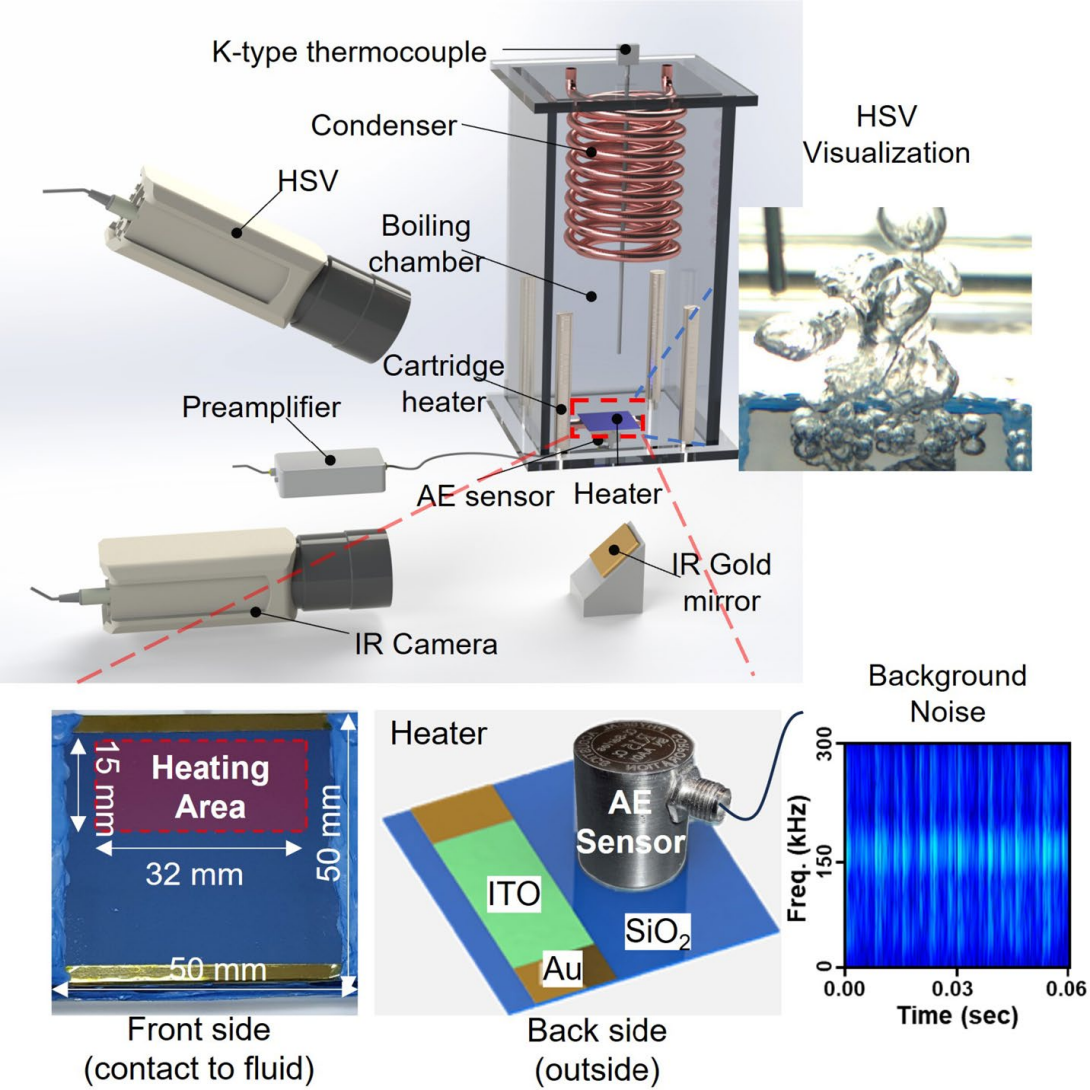
(**a**) Confusion matrix for boiling regime classification (TF-Spec model for flow boiling), and (**b**) Comparison of TF-Spec models for flow boiling prediction.

In boiling regime classification, as shown in the confusion matrix (Fig. 10(a)), all test dataset predictions were 100% accurate across the three boiling regimes (FC, NB, and CHF). This classification performance can be attributed to the reduced noise levels in the flow boiling AE signals compared to those in pool boiling because, unlike pool boiling, there are fewer noise-generating factors such as cartridge heaters. The presence of flow likely suppressed extraneous noise, resulting in cleaner signals that facilitated precise regime identification.

For more detailed results, Fig. 10 (b) summarizes the comparative performance of the three TF-Spec configurations—Transfer Learning, Standalone Training, and Pool Boiling—across three metrics: NRMSE for heat flux and HTC predictions, and classification accuracy for boiling regimes. The Transfer Learning and Standalone Training models achieved nearly identical NRMSE values for both heat flux (0.0437 and 0.0432, respectively) and HTC predictions (0.0443 and 0.0419, respectively), significantly outperforming the Pool Boiling model, which exhibited much higher errors (NRMSE of 0.3526 for heat flux and 0.3063 for HTC).

While the current flow boiling study demonstrates how transfer learning can adapt the TF-Spec model to altered thermal–hydraulic conditions, future work will further examine how different heater geometries, surface coatings, or working fluids might shift AE signal characteristics. Because bubble nucleation dynamics remain the primary source of AE, we anticipate that similar impulse-type signals will persist when operating temperatures and pressures remain comparable. Significantly different working fluids or system conditions may require additional fine-tuning or domain adaptation.

## Conclusion

This study confirms that acoustic emission (AE) signals, when combined with deep learning, can accurately predict key boiling parameters such as heat flux, HTC, and boiling regimes. We developed various models and identified the Transformer-based spectrogram (TF-Spec) as the top performer, achieving ± 20% error margins and over 98% classification accuracy for heat flux, heat transfer coefficients, and boiling regimes under pool boiling. We further validated TF-Spec under flow boiling, which involves additional convective complexities, using both transfer learning and standalone training. Transfer learning effectively leveraged pre-trained knowledge, minimizing retraining while maintaining high accuracy. These findings highlight AE-based monitoring as an appealing solution in non-intrusive boiling system where direct measurements are challenging, reducing reliance on conservative safety margins.

## Methods

### Pool boiling experiment

**Fig. 11:**
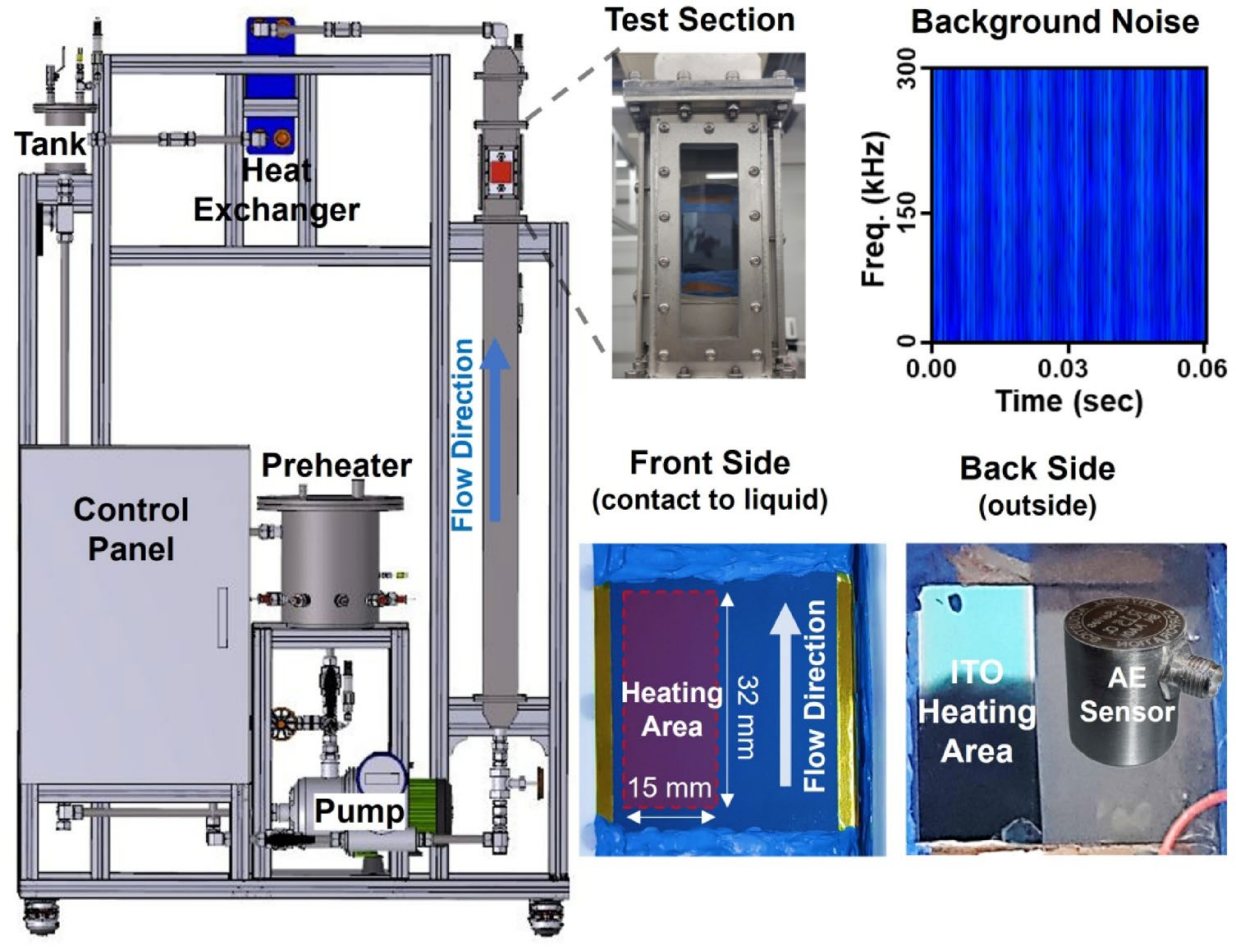
Pool boiling experimental facility for AE signal measurement^21^the configuration of the front side heater and backside of heater with AE sensor, sample image of HSV visualization, and background AE signal noise before conducting experiment.

This study utilized pool boiling experimental data from previous study^21,40^ to investigate boiling acoustic phenomena and develop a deep learning framework. The experimental setup as shown in Fig. 11 consisted of a square flat heater, boiling chamber, power supply unit, standard resistances, condenser, data acquisition system, and acoustic signal measurement instruments. High-speed video (HSV) and infrared (IR) cameras were employed to visualize the boiling process. The boiling chamber, fabricated from transparent polycarbonate, allowed clear observation of the boiling phenomena. Four internal heaters, controlled by a PID system, were installed to maintain the temperature stability of the deionized water used as the working fluid.

Experiments were conducted at atmospheric pressure using a SiO₂/ITO heater with 50 × 50 × 0.5 mm³ as the heating element. The heater comprised an indium tin oxide layer (ITO) with 8.0 Ohm on a silicon substrate with gold electrodes and an insulating SiO₂ layer so that active heating area is 15 mm × 32 mm. Heat flux was controlled through Joule heating on ITO layer by adjusting the supply voltage through gold electrode and the hydrophilic heater surface (static contact angle ~ 41°) supports stable bubble nucleation. Deionized water served as the working fluid, and the boiling chamber was prepared by degassing for 2 h and establishing saturated conditions. In each experiment, the heat flux is incrementally increased in steps—10 kW/m² increments before onset of boiling, then 100 kW/m² increments once nucleate boiling is established—until CHF is reached, beyond which the heater eventually fails.

To visualize bubble behavior, a high-speed video camera (HSV) operating at 3,000 fps and a parallel infrared (IR) camera are used. The IR camera observes the underside of the heater via a gold mirror, converting IR intensities into surface temperature through a separate calibration. Meanwhile, the HSV captures bubble nucleation and departure phenomena within the chamber, synchronized to the AE signal recording with approximately 10 µs accuracy (calibrated via metal-ball impact testing before the experiment). By aligning these two datasets, it becomes possible to correlate transient AE events with specific bubble activities and surface temperature variations.

To measure the AE signals generated during the boiling process, a contact AE sensor (*R15a*,* Physical Acoustics*) was attached adjacent to the heating area on the outer surface of the boiling chamber as shown in Fig. 11 (Cylinder shaped and grey-colored sensor). The sensor captured transient elastic waves produced by bubble nucleation at the heater surface and transferred through heater material. These AE signals contained valuable information related to the boiling dynamics by reflecting the intensity and frequency of boiling events. The high-frequency AE data were collected using a data acquisition system with 1 MHz sampling rate sufficient to capture the detailed acoustic signatures associated with different boiling regimes. By focusing on AE measurements, the study leveraged non-intrusive monitoring to analyze thermal performance, which was essential for developing the deep learning framework aimed at predicting heat flux, heat transfer coefficients, and boiling regimes.

All measured parameters in this study were subject to both instrumental and systematic uncertainties. The key measurement components and their respective ranges and uncertainties are listed in Table 3. To quantify the overall uncertainty of derived quantities (e.g., heat flux and heat transfer coefficient), we applied the standard error-propagation approach based on the partial derivatives of each contributing variable. For heat flux, q′′ was obtained from measured voltages across the heater (V_heater_​) and the standard resistor (V_std_​), as well as the known area of the heater surface (15 mm × 32 mm​) and the resistor value (R_std_​). Using uncertainty propagation, relative uncertainty can be derived by:

$$\:\frac{\varDelta\:q"}{q"}=\sqrt{{\left(\frac{\varDelta\:{V}_{heater}}{{V}_{heater}}\right)}^{2}+{\left(\frac{\varDelta\:{V}_{std}}{{V}_{std}}\right)}^{2}+{\left(\frac{\varDelta\:{R}_{std}}{{R}_{std}}\right)}^{2}+{\left(\frac{\varDelta\:{A}_{heater}}{{A}_{heater}}\right)}^{2}}$$

Also, the heat transfer coefficient is computed by dividing the heat flux q′′ by the difference between the measured heater surface temperature (T_heater_) and the saturation temperature (T_sat_) of working fluid, and uncertainty can be derived by:

$$\:\frac{\varDelta\:HTC}{HTC}=\sqrt{{\left(\frac{\varDelta\:q"}{q"}\right)}^{2}+{\left(\frac{\varDelta\:{T}_{heater}}{{T}_{heater}}\right)}^{2}}$$

From the uncertainty valued in Table 3, the contributions to V_heater_​ and V_std_​ are typically ± 1% from power supply and measurement unit calibration, R_std_ of ± 0.001%, constant A_heater_​, and IR-based temperature measurement uncertainty is ± 1.7%. Based on these values, estimated uncertainty of the resulting heat flux is about ± 1.4% over the heat flux range of 0–5.8 MW/m² and uncertainty of HTC is ± 2.2%. For the acoustic emission (AE) signals, we employed an R15a-type contact AE sensor (MISTRAS Group). Based on the sensor’s directional and frequency-response variability (± 1.5 dB directionality), as well as typical calibrations, the overall amplitude (voltage) uncertainty is estimated at about ± 17.4%.

**Table 3:** The uncertainty of boiling experimental measurement.

Parameter	Instrumentation	Meas. range	Uncertainty
Voltage (V)	Multifunction measure unit with DC power supply	0–150 V	± 1%
Current (A)		0–18.8 A	± 1%
Resistance (Ohm)	Resistance standard	0.001 Ω	± 0.01%
Heat Flux (W/m^2^)	$\:q"=\frac{{V}_{heater}\times\:{V}_{std}}{{A}_{heater}\:\times\:\:{R}_{std}}\:\:$	0–5.8 MW/m^2^	± 1.4%
Heat temperature (℃)	IR Thermography	80 ℃ – 200 ℃	± 1.7%
HTC (W/m^2^K)	$\:HTC=\frac{q"}{{T}_{heater}-{T}_{sat}}$	0–35.0 kW/m^2^K	± 2.2%
AE signal (V)	R15a Contact AE sensor	± 10 V	± 17.4%

**Table 4:** Total dataset of acoustic emission signal from pool boiling experiment.

Boiling Regime	NC	NB	CHF	Total
**Number of Dataset**	3,436	12,795	1,875	18,106
**Heat flux range [kW/m** ^**2**^ **]**	1 ~ 31	40 ~ 932	895 ~ 940	1 ~ 940
**HTC range** **[kW/m** ^**2**^ **K]**	1.1 ~ 3.8	3.6 ~ 30.5	22.2 ~ 29.9	1.1 ~ 30.5

As shown in Table 4, this dataset provides a comprehensive range of AE signals spanning from natural convection (NC) through nucleate boiling (NB) and into the critical heat flux (CHF) regime. In total, it encompasses 18,106 data points, covering heat fluxes from approximately 1 kW/m² under NC conditions up to about 940 kW/m² near CHF, as well as a broad range of heat transfer coefficients (1.1 to 30.5 kW/m²K). Many of the data falls within the nucleate boiling regime, illustrating the complex progression from isolated bubble events to more vigorous boiling activity. By including AE signals from both non-boiling and advanced boiling conditions, this dataset is designed to rigorously test the generalization and robustness of deep learning-based prediction models across diverse and realistic thermal environments. (a portion of datasets are available in *the data availability* section)

### Deep learning methodology

This study aims to develop a technique for monitoring the internal heat flux, HTC, and boiling regimes using AE signals measured externally. To achieve this, we constructed and evaluated a variety of deep learning models, seeking to identify both the optimal input representation (AE signals or spectrograms) and the most suitable model architecture. As shown in Table 5, The deep learning models employed in this study used two types of inputs: the one-dimensional AE signal (61,441 × 1) and the two-dimensional spectrogram (256 × 256). These inputs provided the models with distinct representations of the data, enabling the exploration of both temporal and frequency-domain features for enhanced predictions. The primary prediction targets included heat flux (ranging from 1 to 940 kW/m²), heat transfer coefficient (HTC) (ranging from 1.1 to 30.5 kW/m²K), and boiling regimes, classified into three categories: natural convection (NC), nucleate boiling (NB), and critical heat flux (CHF).

**Table 5:** Input and prediction values with the evaluation methods for pool boiling.

Input	Prediction values	Evaluations
AE Signal (61,441 × 1)	Heat flux: 1 ~ 940 kW/m^2^ HTC: 1.1 ~ 30.5 kW/m^2^K Boiling regimes: - Natural convection (NC) - Nucleate boiling (NB) - Critical heat flux (CHF)	RMSE = $\:\sqrt{\frac{1}{n}{\sum\:}_{i=1}^{n}{\left({y}_{i}-{\widehat{y}}_{i}\right)}^{2}}$ NRMSE = $\:\frac{\sqrt{\frac{1}{n}{\sum\:}_{i=1}^{n}{\left({y}_{i}-{\widehat{y}}_{i}\right)}^{2}}}{{y}_{max}-{y}_{min}}$ Accuracy = $\:\frac{{\sum\:}_{i=1}^{n}I({y}_{i}=\widehat{{y}_{i}})}{n}$
Spectrogram (256 × 256)		

We began by testing numerous hyperparameter combinations for four types of deep learning models – Deep Neural Network (DNN)^22^Convolutional Neural Network (CNN)^22,36^Transformer (TF)^37^and Fourier Neural Operator (FNO)^38^ - using randomly selected 10% of the total dataset to efficiently explore the parameter space. Specifically, as illustrated in Fig. 3, we initially trained and compared a total of 245 different models. Due to the substantial computational time associated with training such a large number of models, we first utilized a smaller subset (10%) of the dataset for preliminary hyperparameter tuning and initial model evaluation. This approach significantly reduced computational costs and allowed efficient identification of promising model candidates. Once the optimal configuration was found for each model, we then retrained and tested the chosen models using the entire dataset, thus ensuring that the results were based on comprehensive data usage. As illustrated in Fig. 3 and summarized in Table 6, four core deep learning approaches were investigated:

- DNN^22^: The one-dimensional AE signal (61,441 × 1) or a flattened spectrogram (256 × 256) served as input. We constructed models with 3 to 6 hidden layers and 10 to 10,000 neurons per layer, systematically testing a wide range of parameter configurations. Each hidden layer applies linear transformations followed by non-linear activations (ReLU), enabling the network to learn hierarchical patterns. Training employed backpropagation, which iteratively adjusts weights to minimize loss functions (Fig. 3(a), Table 6).
- CNN^22,36^: For the convolutional neural network (CNN), we adapted widely used architectures (ResNet-50^41^, AlexNet^42^VGG16^43^, EfficientNet-b4^44^) to suit our application. The AE signal or spectrogram data were reshaped into two-dimensional arrays (256 × 256) to match the input requirements of CNNs. This reshaping allows CNNs to leverage spatial hierarchies in the data, such as local frequency patterns in spectrograms. Each CNN architecture incorporates convolutional layers for feature extraction, pooling layers for dimensionality reduction, and fully connected layers for final predictions. For example, ResNet-50 employs residual connections to mitigate vanishing gradient problems, while EfficientNet-b4 balances depth, width, and resolution for optimized performance. These architectures were chosen for their ability to efficiently capture spatial patterns and their proven performance in similar tasks (Fig. 3(b), Table 6).
- Transformer (TF)^37^: Various configurations were explored to identify optimal use of attention mechanisms for refined feature extraction (Fig. 3(c), Table 6). Specifically, the model dimension (D-model: 32 to 768) determines the size of the embedding input and overall representation capacity of each sequence element. The number of attention heads (2 to 16) influences how the input embeddings are projected into multiple subspaces, enabling the model to capture different types of relationships and patterns in parallel. Adjusting the number of encoder layers (2 to 12) alters the model’s depth, stacking multiple attention and feedforward blocks. The feedforward dimension (128 to 3072) sets the capacity of the fully connected layers within each encoder block, allowing for more complex transformations and enriching the learned feature representations. Together, these parameters control the Transformer’s ability to extract nuanced patterns from AE signals under varying conditions.
- FNO^38^: For the Fourier Neural Operator (FNO) model, we tested a range of parameters to handle only the raw AE signal as input. Unlike the other models, we did not use spectrograms, as they are already Fourier-transformed and may not align optimally with the additional Fourier transformations the FNO performs. In the FNO model, “Modes” represent the number of Fourier modes and directly influence the maximum frequency the model can learn. For example, 1,000 modes capture up to ~ 81.4 kHz, while 3,687 modes can represent frequencies up to ~ 300 kHz. Since our spectrogram data spanned 0–300 kHz, we chose modes between 1,000 and 3,687 to cover the full frequency range. The parameter “Width” defines the number of channels per layer, controlling model capacity (set between 32 and 128), and we varied the number of layers (2 to 4) performing Fourier transformations to optimize performance (Fig. 3(d), Table 6).

To ensure robust and generalizable performance across architecture, we conducted a partial sensitivity analysis on key hyperparameters for all four deep learning models—DNN, CNN, Transformer, and FNO based-on Table 6. For DNNs, we varied the number of hidden layers and neurons per layer to evaluate the impact of model depth and capacity on regression and classification accuracy. This was essential to prevent underfitting with shallow networks or overfitting with excessively large architectures. In CNNs, we compared widely used backbone architectures such as ResNet, VGGNet, AlexNet, and EfficientNet, which vary in depth, convolutional kernel size, and skip connections. This allowed us to assess the optimal balance between spatial feature extraction and model complexity, particularly for spectrogram inputs where spatial frequency features play a key role. For Transformer models, sensitivity analysis focused on attention-specific parameters such as the number of encoder layers, attention heads, hidden dimension size (D-model), and feedforward layer width. These influence how well temporal and frequency-domain dependencies in AE signals are captured, and our analysis helped identify configurations that offer the best trade-off between computational efficiency and learning capacity. In the case of FNO, we tested various numbers of Fourier modes (affecting the maximum frequency captured), network widths, and Fourier transformation layers. Since AE signals are inherently frequency-rich, tuning these parameters was critical to ensure effective learning in the frequency domain without redundancy or excessive model burden.

**Table 6:** Test matrix of deep learning models with architectures and hyperparameters.

Backbone	Architecture		Hyperparameters
DNN	Hidden layers: 3 ~ 6 Neurons per layer: 10 ~ 10,000 Parameters: 307,380 ~ 786,943,500		Activation: Relu Optimizer: Adam Loss: MSE, CorssEntropy Epochs: 100 ~ 400 Patience: 50 Dropout: 0.1 Batch size: 15 ~ 50 Batch normalization Learning rate: 0.01 ~ 0.0001 Scheduler: ReduceLROnPlateau 4-fold cross validation Weight initialization: He
CNN	Resnet-50 AlexNet VGGNet-16 EfficientNet-b4		
Transformer	D-models: 32, 64, 128, 256, 768 Heads: 2, 4, 8, 12, 16 Encoders: 2, 4, 6, 8, 12 D-feedforward: 128, 256, 512, 1024, 3072		
FNO	Modes (Max. Freq.):	1,000 (81.4 kHz) ~ 3,687 (300.0 kHz)	
	Widths: 32, 64, 128 Fourier Layers: 2, 3, 4		

Common hyperparameters included ReLU activation, Adam optimizer, MSE for heat flux and HTC prediction and CrossEntropy loss function for boiling regimes prediction, 100–400 training epochs, and techniques like dropout (rate: 0.1), batch normalization, early stopping (patience: 50), and learning rate schedulers to prevent overfitting. A 4-fold cross-validation strategy was employed, and weight initialization followed the He method. Each deep learning model took either AE signals or spectrograms as input and was tasked with predicting heat flux, HTC, and boiling regime. Models using AE signals as input were designated DNN-Signal, CNN-Signal, TF-Signal, and FNO-Signal, while those using spectrograms were named DNN-Spec, CNN-Spec, and TF-Spec.

All deep learning training and evaluation processes employed a hybrid approach to accommodate the simultaneous regression and classification tasks. For regression targets such as heat flux and HTC, Root Mean Squared Error (RMSE) was used to measure the average prediction error magnitude, providing insight into the model’s accuracy for continuous outputs. Additionally, Normalized Root Mean Squared Error (NRMSE) was employed to normalize RMSE by the target variable’s range, enabling consistent comparisons across datasets with varying scales. For classification tasks like boiling regime predictions, classification accuracy was used to quantify the proportion of correctly classified instances, offering a straightforward measure of model performance.

To evaluate overall model performance across the three prediction outputs, a composite score was calculated as an average of 1 − NRMSE of heat flux, 1 − NRMSE of HTC, and classification accuracy of boiling regimes as follows:

$$\:Score=Average\left[\left(1-NRMS{E}_{HeatFlux}\right)+\left(1-NRMS{E}_{HTC}\right)+Ac{curacy}_{BR}\right]$$

This scoring method balances the regression and classification tasks, ensuring that the model’s ability to predict continuous variables and classify boiling regimes are weighed equally when determining optimal performance. All training, validation, and inference steps were implemented using Python and the PyTorch^45^ library in NVIDIA RTX 3090 environment, ensuring flexibility and efficiency in model development and testing (developed codes are available in the *code availability* section).

### Flow boiling experiment

The flow boiling experiment was conducted using a closed-loop system designed to investigate vertical upward flow boiling and to measure AE signals generated during the process, as shown in Fig. 8. The loop consisted of a water tank, centrifugal pump, preheater, test section, and heat exchanger, connected by 3/4-inch SS316 stainless steel tubing. The preheater, located upstream of the test section, served to regulate the inlet temperature via PID control and provided flow stabilization. The test section comprised a 35 × 70 mm^2^ SS316 rectangular duct with a side-mounted transparent quartz glass window, allowing flow visualization using HSV. Experiments were performed under atmospheric pressure conditions with deionized water as the working fluid, at an inlet subcooling of 25 K and a flow flux of 165 kg/m^2^s (25 LPM).

A SiO_2_/ITO heater was installed inside the test section to supply controlled heat flux through resistive Joule heating. The surface temperature was measured using an IR camera combined with IR intensity-temperature calibration. Boiling behavior was recorded visually and categorized into three regimes: single-phase forced convection (FC), nucleate boiling (NB), and critical heat flux (CHF). CHF was defined as the onset of irreversible dryout, when the dry patch on the heater surface no longer collapses but continues to grow. AE signals were recorded using the same R15a (MISTRAS) broadband contact-type sensor attached directly to the heater surface to capture the elastic waves generated during the boiling process.

**Table 7:** Total dataset of acoustic emission signal from flow boiling experiment.

Boiling Regime	FC	NB	CHF	Total
**Number of Dataset**	100	738	14	852
**Heat flux range [kW/m** ^**2**^ **]**	0 ~ 50	100 ~ 2,000	2,050	0 ~ 2,050
**HTC range [kW/m** ^**2**^ **K]**	0.0 ~ 3.3	4.8 ~ 25.0	24.5	0.0 ~ 25.0

The collected AE signals were processed and labeled based on synchronized HSV observations to classify the corresponding boiling regimes. A total of 852 data samples were obtained from the flow boiling experiments, covering a broad range of thermal conditions. As summarized in Table 7, the dataset includes 100 samples from the forced convection (FC) regime, 738 samples from the nucleate boiling (NB) regime, and 14 samples from the critical heat flux (CHF) regime. The associated heat flux values ranged from 0 to 2,050 kW/m², while the corresponding heat transfer coefficients (HTC) ranged from 0.0 to 25.0 kW/m²K. This flow boiling dataset was subsequently used to evaluate the generalizability and robustness of the deep learning models trained on the pool boiling data.

There were notable differences in the background noise of AE signals between flow boiling and pool boiling. In pool boiling, a pronounced 150 kHz peak was observed in the background noise, which we attributed to the heating of cartridge heaters in the surrounding area, as confirmed by examining the on/off noise patterns. In contrast, although flow boiling exhibits some pulsation due to the centrifugal pump motor operation, this noise component is not nearly as dominant.

## Data Availability

A portion of the datasets generated and analyzed during this study is available on GitHub (https://github.com/TH-dyLIM/Decoding-the-Whispers-of-Boiling). The full dataset is available from the first author, D. Lim (dylim@tamu.edu), or the corresponding author, I.C. Bang (icbang@unist.ac.kr), upon reasonable request.

## Abbreviations

AE: Acoustic Emission
BR: Boiling Regime
CHF: Critical Heat Flux [kW/m^2^]
CNN: Convolutional Neural Network
DNN: Deep Neural Network
FNO: Fourier Neural Operator
FFT: Fast Fourier Transform
HTC: Heat Transfer Coefficient [kW/m^2^K]
HSV: High-Speed Visualization
IR: Infrared
ITO: Indium Tin Oxide
MLP: Multi-Layer Perceptron
NB: Nucleate Boiling
NC: Natural Convection
NRMSE: Normalized Root Mean Squared Error
RMSE: Root Mean Squared Error
STFT: Short Time Fourier Transform
TF: Transformer
A: Area of Heater [m^2^]
R: Resistance [ohm]
T: Temperature of Heater or Fluid [℃]
V: Applied Voltage [V]
n: Number of Samples
${\rm q''}$: Heat Flux [kW/m^2^]
$y_{i}$: Ground-truth (true) Value of the i-th Sample
$\hat{y}_{i}$: Predicted Value of the i-th Sample
